# The evolutionary dynamics of biological invasions: A multi‐approach perspective

**DOI:** 10.1111/eva.13215

**Published:** 2021-03-30

**Authors:** Stéphanie Sherpa, Laurence Després

**Affiliations:** ^1^ CNRS LECA Université Grenoble Alpes Université Savoie Mont Blanc Grenoble France

**Keywords:** biological invasion, demo‐genomics, dispersal, genetic diversity, insect, integrative approach, local adaptation, occurrence time series, species distribution modeling

## Abstract

Biological invasions, the establishment and spread of non‐native species in new regions, can have extensive economic and environmental consequences. Increased global connectivity accelerates introduction rates, while climate and land‐cover changes may decrease the barriers to invasive populations spread. A detailed knowledge of the invasion history, including assessing source populations, routes of spread, number of independent introductions, and the effects of genetic bottlenecks and admixture on the establishment success, adaptive potential, and further spread, is crucial from an applied perspective to mitigate socioeconomic impacts of invasive species, as well as for addressing fundamental questions on the evolutionary dynamics of the invasion process. Recent advances in genomics together with the development of geographic information systems provide unprecedented large genetic and environmental datasets at global and local scales to link population genomics, landscape ecology, and species distribution modeling into a common framework to study the invasion process. Although the factors underlying population invasiveness have been extensively reviewed, analytical methods currently available to optimally combine molecular and environmental data for inferring invasive population demographic parameters and predicting further spreading are still under development. In this review, we focus on the few recent insect invasion studies that combine different datasets and approaches to show how integrating genetic, observational, ecological, and environmental data pave the way to a more integrative biological invasion science. We provide guidelines to study the evolutionary dynamics of invasions at each step of the invasion process, and conclude on the benefits of including all types of information and up‐to‐date analytical tools from different research areas into a single framework.

## 1 INTRODUCTION

Biological invasions are becoming a common feature of ecosystems worldwide and are recognized as one of the main driver of biodiversity changes in the recent time (Mollot et al., 2017; Seebens et al., 2017). The impact of introduced species on invaded communities and ecosystem functioning is an ever‐growing concern out of the scope of the present review, which focuses on population processes to show that there is much to gain in integrating various types of information (historical, genetic, environmental, phenotypic) and of approaches (empirical population studies, laboratory experiments, modeling) in invasion studies. The many demo‐genetic processes involved during the invasion process have been extensively reviewed during the last 20 years (Table S1) and are summarized in Box 1. They include the many facets of the introduction modalities, such as the origin of populations, the number of independent introductions, and admixture/hybridization, and the ecological characteristics of the niche, such as climatic conditions, landscape features, and biotic interactions, that will constitute the selective landscape faced by introduced populations.

BOX 1 **Demo‐genetic and evolutionary processes during biological invasion**

Species that succeed in establishing and expanding outside their initial range have long been viewed as a genetic paradox (Sax & Brown, 2000). Yet, this view is currently challenged by the interplay between genetic, demographic, and environmental factors shown to underlie the invasion success (Bock et al., 2015; Estoup et al., 2016; Facon et al., 2006). The impacts of these factors on the genetic variability within and between invasive populations and their source(s), and their role in the establishment or expansion of invasive species have been largely reviewed (Table 1 and S1). Population processes are governed by spatial and temporal patterns. Their understanding therefore requires a good knowledge of the invasion history (relevant genetic units to compare).

**TABLE 1:** Processes explaining the genetic diversity of introduced populations and the genetic differentiation among invasive populations or between invasive populations and their source(s)

Term	Description	Consequence
Founder effect	Subsampling of the gene pool of the source population increasing the probability to undergo genetic bottleneck (increased influence of genetic drift) (Sakai et al., 2001)	Reduced genetic diversity in the introduced population and change in allelic frequencies between the introduced population and its source
Genetic bottleneck	Loss of low‐frequency alleles due to genetic drift resulting in an inbreed progeny accumulating recessive deleterious mutations (genetic load)	Reduced genetic diversity and mean fitness (inbreeding depression (Charlesworth & Willis, 2009)) of the introduced population
Purge of genetic load	Purge of homozygous deleterious alleles reducing genetic load (Glémin, 2003)	Increased fitness of the genetically reduced introduced population (bottleneck of intermediate intensity) compared to its source
Expansion load	Increased deleterious genetic diversity in expanding wave fronts due to successive bottlenecks (spatial pattern) during expansion (Peischl & Excoffier, 2015)	Reduction of genetic diversity in edge populations compared to their source (core) and progressive differentiation due to the stochastic fixation of alleles (allele surfing (Excoffier & Ray, 2008))
Multiple introductions (temporal)	Several independent introduction events at the same location (Dlugosch & Parker, 2008)	See propagule pressure for multiple introductions from one source, and admixture for multiple introductions from several sources
Multiple introductions (spatial)	Several independent introduction events at different locations (independent histories) (Dlugosch & Parker, 2008)	Genetic differentiation between introduced populations results from distinct origin (multiple sources) or demographic features (one source)
Propagule pressure	Introduction effort at one location from one source: number of individuals in each introduction event (propagule size) and number of introduction events (propagule number) (Lockwood et al., 2005)	Propagule size determines the founding genetic diversity and propagule number maintains initial genetic diversity by reintroducing alleles lost through genetic drift
Admixture/Hybridization	Interbreeding following independent introduction events at one location (temporal pattern) or secondary contact between expanding populations (spatial pattern) within species or between introduced populations and another species	Increased genetic diversity that can alleviate the negative effects of bottlenecks through the masking of genetic load (heterosis) and create novel genetic combinations with new combinations of traits (neutral introgression) (Mesgaran et al., 2016; Rius & Darling, 2014)
Bridgehead effect	Successful invasive populations in any geographic area serve as the source for new introductions (Lombaert et al., 2010), both at intra‐ and at intercontinental scales	Self‐accelerating invasion process: advantageous changes at the bridgehead population further increase invasiveness (Bertelsmeier & Keller, 2018)
Preadaptation	Introduced population retained the ecological niche of its source (niche conservatism) and phenotypic traits required to invade preexist in the source population (Hufbauer et al., 2012; Mack, 2003)	Introduced population diversity depends on the demographic features during introduction but adaptive traits depend on the history of the source population
Post‐introduction adaptation	The introduced population displays evidence of rapid adaptation in response to any change in the environmental niche (niche shift) of its source (Lee, 2002; Prentis et al., 2008)	Both diversity and adaptive traits depend on the demographic features during introduction. Propagule pressure and admixture/hybridization increase genetic variance that natural selection can act upon (compared to *de novo* mutation)
Adaptive introgression	Gene flow (admixture/hybridization) provides functional adaptive alleles that are incorporated in the gene pool of the recipient introduced population (Largiadèr, 2008)	Introduced population diversity depends on the demographic features during introduction but adaptive traits are inherited from the donor taxa
Spatial sorting	Increased dispersal abilities in low‐density inbred populations as the result of natural selection during expansion (Phillipds & Perkins, 2019)	Acceleration of the expansion speed despite lower genetic diversity in expanding populations than in its source (core)

The genetic characteristics of invasive populations can reflect neutral processes such as (i) ancestral divergence of native lineages, (ii) demo‐genetic dynamics during each step of the invasion process (founder effects during introduction, allele surfing during expansion), and (iii) population connectivity (current gene flow), and/or adaptive processes such as (iv) preadaptation within the native or bridgehead invasive source populations, and (v) postintroduction adaptations (adaptation during establishment, spatial sorting during expansion, adaptive introgression following admixture/ hybridization events) (Box 1). Because the complex demographic processes and evolutionary adaptive processes are simultaneously at play, disentangling their respective roles during the invasion process is particularly challenging (Keller & Taylor, 2008). The recent development of high‐throughput sequencing (HTS) technologies provides unprecedented high‐quality genomic datasets to simultaneously address these challenges, as both neutral variation and adaptive variation are accessible from whole genomes or their simplified representation. Furthermore, the large amount of information available from a single genome allows to precisely measure the genomic composition at the individual scale, with no need to sample many individuals per population to have access to key population genetics parameters such as inbreeding coefficients or genetic structure indices, which is particularly appreciable when dealing with invasive populations (McCartney et al., 2019; Rius et al., 2015).

Genome‐scale datasets have triggered the development of new analytical methods able to deal with large amount of information. Here, we provide an overview of these methods for addressing (i) population structure and colonization routes, (ii) demographic inferences, (iii) local adaptation, and (iv) landscape genetics, the type of data used and the software names, and their utility and their limits (Table 2; Appendix S1). These methods are either based on probabilistic models, summary statistics, and intensive computational simulations (e.g., ABC methods), or on model‐free ordination approaches (e.g., PCA). These flexible analytical approaches provide detailed information on population genetic composition, structure, and demographic parameters. They allow testing alternative demographic scenarios at all the spatiotemporal scales required to study complex invasion histories, from introduction modalities to spread and local adaptation. Only some of these methods explicitly integrate geospatial datasets in addition to genomic datasets to determine landscape connectivity or to link adaptive and environmental variation.

**TABLE 2:** Nonexhaustive list of methods used for examining evolutionary processes in the context of biological invasions based on genetic data. More details on the use and limits are provided in Appendix S1

Use to detect/infer	Software implementation	Used information/index	Models/approach	Type of data^a^	Reference
**Population structure/Colonization routes**					
Phylogenetic relationships	RAxML	Phylogenetic trees	Maximum likelihood	Multiple sequence alignment	Stamatakis et al. (2008)
Genetic structure of populations	R (hierfstat) – *F*_ST_	*F*_ST_	Frequencies‐based model	Diploid multilocus genotypes	Goudet (2005)
	Arlequin – AMOVA	*F*_ST_	Genetic variance partitioning	Multiple sequence alignment Diploid multilocus genotypes	Excoffier et al. (2005)
	STRUCTURE	Assignment	Frequencies‐based model	Diploid multilocus genotypes	Pritchard et al. (2000)
	R (LEA) – SNMF	Assignment	Frequencies‐based model	SNPs	Frichot et al. (2014)
	ADMIXTURE	Assignment	Frequencies‐based model	SNPs	Alexander et al. (2009)
	R (adegenet) – DAPC	Assignment	Ordination methods (PCA + DA)	Diploid multilocus genotypes	Jombart et al. (2010)
Migration rates	GeneClass2	Assignment	Frequencies‐based model	Diploid multilocus genotypes	Piry et al. (2004
	BayesAss	Assignment	Frequencies‐based model	Diploid multilocus genotypes	Wilson and Rannala (2003)
	MIGRATE	Assignment	Frequencies‐based model	Multiple sequence alignment Diploid multilocus genotypes	Beerli (2002
Source populations	DIYABC	Posterior probability of scenarios	Coalescent backward model + ABC	Multiple sequence alignment Diploid multilocus genotypes	Cornuet et al. (2008)
**Demographic inferences**					
Genetic diversity and bottleneck	DnaSP	*H*_E_, AR, *H*d; Tajima's D, Fu's F, Fu & Li's F	Frequencies‐based model	Aligned sequences	Rozas et al. (2003
	R (hierfstat) – *F*_IS_	Coefficient of consanguinity	Frequencies‐based model	Diploid multilocus genotypes	Goudet (2005)
	BOTTLENECK	*H*_E_ excess, allele frequency distribution, “M‐ratio” test	Frequencies‐based model	Diploid multilocus genotypes	Piry et al. (1999
Changes in effective population size over time	DIYABC	Posterior probability of scenarios	Coalescent backward model + ABC	Multiple sequence alignment Diploid multilocus genotypes	Cornuet et al. (2008)
	dadi	Folded or unfolded SFS	Diffusion approximation	SNPs	Gutenkunst et al. (2009)
	Stairway plot	Folded or unfolded SFS	Coalescent backward model	SNPs	Liu and Fu (2015)
	PopSizeABC	Folded SFS + Recombination (LD)	Coalescent backward model	SNPs	Boitard et al. (2016)
	PSMC	Mapped sequences + Recombination (LD)	Coalescent backward model	Whole‐genome haplotypes (phased)	Li and Durbin (2011)
	MSMC	Mapped sequences + Recombination (LD)	Coalescent backward model	Whole‐genome haplotypes (phased)	Schiffels and Durbin (2014)
	SMC ++	Folded SFS + Recombination (LD)	Coalescent backward model	Whole‐genome haplotypes/SNPs (phased)	Terhorst et al. (2017))
Admixture/Hybridization events	PCAdmix	Local ancestry inference	Ordination method (PCA)	Whole‐genome haplotypes/SNPs (phased)	Brisbin et al. (2012)
	TreeMix	Phylogenetic trees and ancestry coefficients	Maximum likelihood	Diploid multilocus genotypes	Pickrell and Pritchard (2012)
	RFMix	Local ancestry inference	Random Forest	Whole‐genome haplotypes (phased)	Maples et al. (2013)
	Loter	Local ancestry inference	Nearest‐Neighbor approach	Whole‐genome haplotypes (phased)	Dias‐Alves et al. (2018)
	AdmixTools	D (ABBA‐BABA), f‐statistics	Topology tests	SNPs	Patterson et al. (2012)
	Comp‐D	D (ABBA‐BABA), f‐statistics	Topology tests	Whole‐genome haplotypes (phased)	Mussmann et al. (2019)
**Adaptive genomics**					
Putative adaptive loci	BayeScan	*F_ST_* outliers	Frequencies‐based model	Allele frequencies	Foll and Gaggiotti (2008)
	R (OutFLANK)	*F*_ST_ outliers	Frequencies‐based model	SNPs	Whitlock and Lotterhos (2015)
	R (pcadapt)	PC outliers	Ordination method (PCA)	SNPs, allele frequencies	Luu et al. (2017)
Putative adaptive loci showing association with environment	BayeScEnv	Genotype–environment association	Bayesian method (univariate)	Allele frequencies + one environmental/phenotypic variable	De Villemereuil and Gaggiotti (2015)
	BayPass	Genotype–environment association	Bayesian method (univariate)	Allele frequencies + one environmental/phenotypic variable	Gautier (2015)
	R (LEA) – LFMM	Genotype–environment association	Bayesian method (univariate)	Allele frequencies + one environmental/ phenotypic variable	Frichot et al. (2013)
Variance at putative adaptive loci constrained by environment	R (rdadapt)	Genotype–environment association	Ordination method (multivariate)	SNPs +environmental + geographic datasets	Capblancq et al. (2018)
	R (gdm)	Genotype–environment association	Generalized dissimilarity modeling	Genetic +environmental + geographic distance matrices	Manion et al. (2017)
**Landscape genetics**					
Isolation by distance or resistance	R (vegan) – mantel	Dissimilarity‐based analysis	Mantel tests	Geographic/resistance +genetic distance matrices	Oksanen et al. (2007)
	R (ecodist) – MRM	Dissimilarity‐based analysis	Multiple regression	Geographic/resistance +genetic distance matrices	Lichstein (2007)
Isolation by barriers	SPLATCHE 3	Spatial genetic structure	Spatially explicit coalescent model	Georeferenced multilocus genotypes	Currat et al. (2019)
	Geneland	Spatial genetic structure	Map of population membership	Georeferenced multilocus genotypes	Guillot et al. (2005)
	LocalDiff	Spatial genetic structure	Bayesian kriging	Georeferenced multilocus genotypes	Duforet‐Frebourg and Blum (2014)
	EEMS	Spatial genetic structure	Spatially explicit model	Georeferenced multilocus genotypes	Petkova et al. (2016)
Effective resistance distance matrix	PATHMATRIX	Resistance surface (single)	Least‐cost path	Geospatial vector + points	Ray (2005)
	Circuitscape	Resistance surface (single)	Electrical circuit	Geospatial vector + points	Shah and McRae (2008)
	ArcGIS – CostDistance	Resistance surface (single)	Least accumulative cost distance	Geospatial vector + points	ArcGIS (ESRI)
	R (gdistance) – commuteDistance	Resistance surface (single)	Random walk (graph theory)	Geospatial vector + points	van Etten (2012)
	R (ResistanceGA) ‐ GA.prep	Resistance surface (composite)	The algorithm used for each surface	Resistance surfaces to combine	Peterman (2014)

Abbreviations: ABC, approximate Bayesian computation; AR, allelic richness; DA, discriminant analysis; *H*d, haplotype diversity; *H*_E_, heterozygosity; LD, linkage disequilibrium; PCA, principal component analysis; SFS, site frequency spectrum; SNP, single nucleotide polymorphism.

^a^ Diploid multilocus genotypes: both microsatellites, RFLPs, SNPs; otherwise specified.

While HTS was revolutionizing population genomics, geographic information systems were revolutionizing ecology. Species distribution models (SDM, Guisan & Thuiller, 2005) combine known occurrence records with digital layers of environmental variables to assess potentially suitable area for a given species and to predict its future distribution at broad scale. At a more local scale, landscape genetics combines landscape features with spatial genetic variation to infer population connectivity and identify barriers to dispersal (Manel et al., 2003). More direct approaches to study establishment and spread (e.g., population growth, dispersal) involve tedious mark–recapture–release (MRR) experiments and/or experimental designs such as performance measures that cannot be undertaken easily with all species. Nonetheless, HTS approaches such as environmental DNA and metabarcoding can also be useful to study the distribution and spread dynamics of invasive species (Piper et al., 2019). Although these genomic, ecological, and experimental approaches have been undertaken in different studies, none have included all these sources of information into a single framework. This framework should also be able to infer the evolutionary history of a biological invasion and all the associated demo‐genetic parameters: origin(s) and date(s) of introduction(s), colonization route(s), propagule pressure, admixture, changes in population size, and dispersal speed, and to identify environmental parameters acting as barriers/corridors, as well as adaptive traits and genes that underlie invasion success.

Here, we provide an overview of the demo‐genetic and adaptive processes involved at each step of biological invasions, highlighting the approaches and type of information that are needed to fully describe the invasion process, including historical records (detection time series), demographic inferences (source populations, colonization routes, changes in population size overtime), phenotypic measures (fitness traits, dispersal abilities), environmental characterization of source versus invaded areas (niche comparisons), identification of traits and genes involved in adaptation to the novel environment, and their evolutionary history (*de novo* mutations, pre‐adaptations, admixture/hybridization). We discuss the limits and advantages of each of these approaches, from data collection to analytical methods, highlight their complementarity, and identify gaps in our knowledge that need to be filled in order to reach a fully integrative understanding of biological invasion. We structured our review according to the chronological steps of the invasion process although we are aware that assigning the invasive steps can be challenging or may vary in space for a given species.

Among the most invasive taxa, insects represent one of the main threats to socioeconomic systems, as they can have strong impacts on forest and crop productions and on domestic animals and human health. As a consequence, insect invasions are among the most documented invasive systems (Garnas et al., 2016; Kirk et al., 2013; Liebhold & Tobin, 2008; Renault et al., 2018; Roques et al., 2016), with in some cases a good knowledge of the history of introduction and spread thanks to dedicated monitoring structures (e.g., public or private health agencies, crop production companies). In addition to detailed occurrence datasets, phenotypic variation and fitness components can be measured in field or controlled experimental settings relatively easily given the small size and generation time of insects. We take advantage of the large body of literature available on insect invasions and on a few particularly well‐studied cases to illustrate how combining different datasets and approaches can substantially improve our understanding of the invasion process. We focus on insect invasions as model systems but the proposed framework can be applied to any biological invasion.

## 2 IDENTIFICATION OF SOURCE POPULATIONS

The identification of source populations and colonization routes is the first and key step to perform in order to be able to address all the following steps of the invasion process (Estoup & Guillemaud, 2010). Introduced populations are expected to be genetically closer to their source than to any other population. Thus, the source of introductions has been classically deduced using population genetics methods (Excoffier & Heckel, 2006 for a review; Table 2). However, these methods do not account for demographic stochasticity (bottleneck) and complex introduction histories (multiple introductions and admixture) (Estoup & Guillemaud, 2010; Fitzpatrick et al., 2012; Appendix S1). Coalescent modeling coupled with ABC allows testing for different introduction scenarios, involving population split, size changes (bottleneck, expansion) and admixture events, and their timing. This framework is the most commonly used for reconstructing invasion routes nowadays (Garnas et al., 2016 and references therein; Fraimout et al., 2017; Lesieur et al., 2019; Sherpa, Blum, Capblancq, et al., 2019), but requires a rigorous design of the sampling, of alternative scenario topologies and of prior parameters.

### 2.1 Potential sources based on genetic data

**FIGURE 1:**
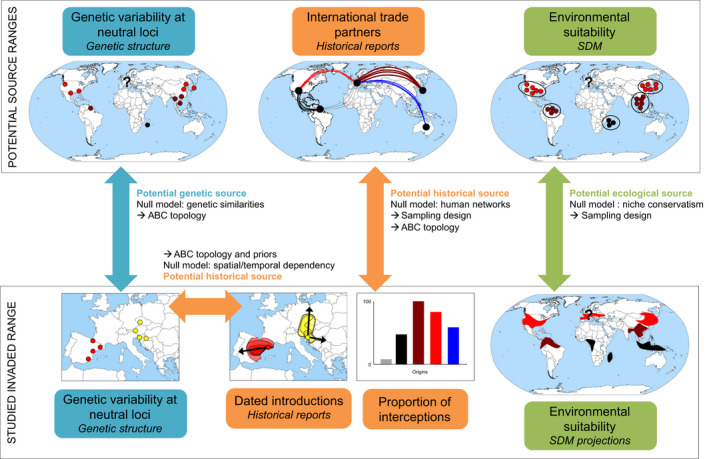
Types of information needed to identify the potential source populations: genetic, historical, and ecological. Blue: genetic variability, orange: dated introductions, green: observational and environmental data. For the illustration, the native range of the species is North America. There are five invaded ranges: Africa, Asia, South America, Australia, and Europe, and the studied invaded area is Europe. ABC scenario topologies are designed based on genetic hypotheses only: genetic similarities among populations (blue) from a representative sampling of the whole distribution range or a reduced sampling based on the likelihood of introduction (orange, no sampling in South America due to low proportion of interceptions) and/or establishment (green, sampling restricted to North America and Asia because predicted invaded ranges from these populations include Europe)

The accurate estimation of the number of introduction events and identification of source populations strongly rely on the sampling design within native and invasive ranges (Cristescu, 2015; Estoup & Guillemaud, 2010). Ideally, sampling should be representative of the whole native range and should include not only the studied invaded area but also all previously invaded areas in order to consider bridgehead scenarios (Lombaert et al., 2010). Introduced populations may be genetically identical to their source especially when introduction is recent or when propagule pressure is high (null model). The genetic differentiation between the focal population and all potential sources can reflect the introduction modalities, such as founder effect and subsequent changes in genetic variation over time through genetic drift and multiple introductions. Thus, the less the time elapsed between first detection and sampling, the higher the probability of identifying the precise source population (Geller et al., 2010). Sampling intensity also influences the probability of identifying the source population (Cristescu, 2015; Geller et al., 2010). Broadscale phylogeographic patterns provide the required knowledge for designing the adequate sampling and alternative scenario topologies (Figure 1). However, the native and invasive areas can be very large especially for human‐mediated invasions, leading to two methodological issues.

The first lies on the existence of a large number of genetically distinct populations. ABC methods require to pool individuals into genetically homogeneous samples between which the evolutionary relationships are tested. It is therefore advisable to first use classical population genetics methods to properly define the genetic samples to be used for demographic inferences (Lombaert, Guillemaud, et al., 2014). Given the large number of plausible introduction scenarios, conducting a step‐by‐step analysis guided by genetic structure is recommended in order to limit the number of scenarios being simultaneously tested (Kerdelhué et al., 2014; Ryan et al., 2019; Sherpa, Blum, Capblancq, et al., 2019). The second consequence is the difficulty to explore exhaustively the ranges outside the studied area. When not much is known about the origin of an invasion or when the true source has not been sampled, including an unsampled ‘ghost population’ is particularly useful (Estoup & Guillemaud, 2010). The construction of complex scenarios involving ghost populations should be done with caution because past changes in effective population size (Ne) should not exceed the time frame allowed by priors (based on time since introduction and mutation rate). A thorough evaluation of the quality of the models and conducting independent ABC analyses on several sample sets can give confidence in the results (Cornuet et al., 2010; Lombaert, Guillemaud, et al., 2014; Appendix S1). Finally, hybridization of the introduced species with local native related taxa can make the detection of the source population more challenging and can have major consequence on the genetic variability and invasion dynamics of the introduced populations. These consequences vary with the strength of the reproductive barrier, which may actually be weaker than previously thought, as exemplified by hybridization between introduced and native taxa that have diverged in allopatry. There are numerous examples of these anthropogenic hybridizations both in introduced plants and animals (Ellstrand & Schierenbeck, 2000; McFarlane & Pemberton, 2019).

### 2.2 Potential sources based on historical records

Occupancy‐detection data represent an important source of information for reconstructing colonization routes (Estoup & Guillemaud, 2010) but not yet fully exploited in genetic studies. Occupancy‐detection data include dated introductions (first detection of the species) and observational data without knowledge of the first occupancy date (occurrences). Occurrence data inform about the distribution ranges and can help designing a representative molecular sampling. For a few species having economic or public health impact, monitoring programs provide high‐quality detection time‐series datasets from early introductions to further expansion (Figure 1). For example, dated introductions were coupled with genomic data to reconstruct the European invasion history of the Asian tiger mosquito *Aedes albopictus* (Sherpa, Blum, Capblancq, et al., 2019). The spatial expansion of the species was inferred from geographically plausible scenarios (i.e., the most likely origin is in geographically close previously invaded areas) that incorporate the dates of introduction as priors in the ABC analysis. Unfortunately, well‐documented historical records are not available for most invasive species.

Historical records can also provide hypothetical routes of colonization and identify potential historical sources prior to molecular sampling (Figure 1). For example, using only ant interceptions at airports and seaports between 1914 and 2013, Bertelsmeier et al. (2018) showed that a large number of ant species have probably been introduced according to a bridgehead scenario. Recent reconstructions of insect invasion histories shed light on the central role of international trade networks in biological invasions. For instance, *A*.* albopictus*, *Drosophila suzukii*, and *Harmonia axyridis* share the same bridgehead invasion scenario, from Eastern Asia to North America (NAM) and then to Europe (Fraimout et al., 2017; Lombaert, Guillemaud, et al., 2014; Sherpa, Blum, Capblancq, et al., 2019). Data on freight routes (e.g., the Port Information Network database for the United States, Mccullough et al., 2006) could be used to estimate the likelihood of introduction from an area based on trans‐ or continental trade partners, especially when the mode of transportation is known (e.g., crops, ornamental plants or tires traders, biocontrol strains).

### 2.3 Potential sources based on ecological niches

Ecological similarities encountered by different and geographically isolated populations can select for similar evolutionary solutions. Thus, fitness‐related traits that evolved in response to ecological constraints in a given geographic area should allow populations to perform well in all the ecologically similar regions they do not yet occupy (i.e., niche conservatism; Wiens & Graham, 2005). Neutral genetic variation does not provide any information on the niche occupied by populations. In biological invasions, SDM constitutes a powerful method to predict species potential distribution and to anticipate invasion risks (Peterson, 2003). Observational data could thus be used to assess the overlap between the distribution ranges predicted by several models: one calibrated using occurrences in the invaded area and a series of models calibrated using occurrences in each potential ecological source. This approach can be applied without prior knowledge on colonization routes but requires describing the spatial distribution of neutral genetic variability among all potential sources (Figure 1). The width of the range overlap gives an indication of the probability of establishment of each potential ecological source under the niche conservatism hypothesis (null model). Incorporating historical data such as transportation networks and the environmental characteristics in two connected areas can also provide information on the probability of introduction and establishment (Tatem & Hay, 2007). The molecular sampling used for reconstructing colonization routes and identify potential genetic sources should (at least) include all the potential source areas where populations have a high probability of establishment in the studied invaded area (historical and ecological sources).

## 3 THE IMPACT OF INTRODUCTION MODALITIES

Understanding the impact of introduction modalities on invasion success requires to (i) identify the most likely source population(s) (colonization routes), (ii) describe the introduction dynamics: levels of genetic diversity in introduced populations and their source(s) or bottleneck intensity (demographic inferences), and (iii) examine the relationship between introduction modalities, the changes in demographic features, and the successful establishment (phenotypic experiments) (Box 2).

BOX 2 **The demo‐genetics of *Harmonia axyridis* invasions**

**Routes of colonization**

**FIGURE 2:**
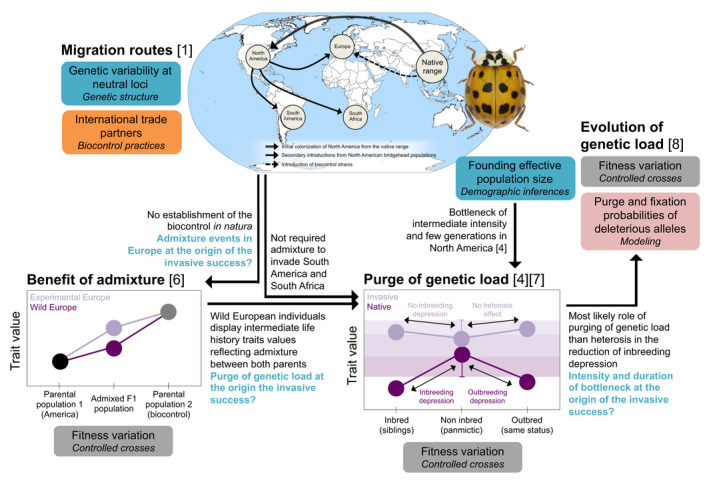
Types of information needed to reconstruct and test the role of introduction modalities in the invasion success using studies in *H*.* axyridis*. Blue: genetic variability, orange: historical data, gray: phenotypic data, and light red: simulated data. Photo from https://commons.wikimedia.org/wiki/File:Harmonia_axyridis_(Pallas_1773).png (CC BY‐SA 4.0). Figures adapted from cited literature. References [1] Lombaert et al. (2010); [2] Blekhman et al. (2020); [3] Lombaert, Guillemaud, et al. (2014)); [4] Facon, Hufbauer, et al. (2011)); [5] Facon, Crespin, et al. (2011)); [6] Turgeon et al. (2011); [7] Tayeh et al. (2013); [8] Laugier, (2013).

From its native Asian range, the Arlequin ladybird *H*.* axyridis* has been intentionally introduced for biological control establishing wild populations in America, Europe, and Africa. Historical data on biocontrol practices and the reconstruction of colonization routes from genetic data demonstrated a bridgehead scenario, involving the colonization of NAM from both Eastern and Western Asia (admixture event 1) that served as the source for the South American (SAM), South African (SAF), and European (EU) invasions (Lombaert et al., 2010). European invasive populations display traces of admixture between NAM and a biological control strain (BIO) used in Europe (admixture event 2) (Figure 2).

**Demo‐genetic dynamics of the introduction**

Haplotype diversity estimated from single DNA locus (mitochondrial DNA) reveals a reduced number of haplotypes among invasive populations as compared to the native ones (Blekhman et al., 2020) confirming their most likely same NAM origin (Blekhman et al., 2020; Lombaert et al., 2010). Diversity estimates from multilocus microsatellites support the role of introduction modalities with lower allelic richness in single introductions from NAM (SAM and SAF) and higher expected heterozygosity in EU than in both parents (BIO and NAM) (Lombaert, Guillemaud, et al., 2014). Based on the topology of introductions (Facon, Hufbauer, et al., 2011) computed ABC analysis to infer the demographic parameters during introduction in NAM and found a bottleneck of intermediate intensity (150 individuals and 20 generations).

**Relationship between introduction modalities and establishment success**

Colonization routes and demographic inferences were used to recreate (controlled crosses) the introduction history and better understand the role of admixture (heterosis) and inbreeding (purge of genetic load). F1 hybrids display higher (Facon, Crespin, et al., 2011) or intermediate (Turgeon et al., 2011) scores for fitness traits (e.g., developmental time, body length) as compared to parental ones (Figure 2). The experimental and wild EU populations exhibit different trait values, suggesting no fixed heterosis (Turgeon et al., 2011). Successful introductions in other continents (SAM and SAF) only involved a single NAM source (Lombaert et al., 2010). Further controlled crosses performed to evaluate offspring performance from within‐ (inbred) and between‐ (outbred) population crosses found evidence for reduced inbreeding depression in invasive populations (Facon, Hufbauer, et al., 2011; Tayeh et al., 2013). Introduction modalities in NAM correspond to the most favorable conditions for the purging of genetic load through drift, while strong bottlenecks most of the times lead to fixation of deleterious alleles (Laugier, 2013).

### 3.1 Introduction modalities based on demographic inferences

A large number of methods have been developed to reconstruct the demographic history of populations from genetic data (Salmona et al., 2017). In invasion genetics, population demographic parameters have been classically measured by population genetic diversity indices and testing the deviation from mutation‐drift equilibrium (Peery et al., 2012; Table 2). The ABC methods allow estimating current Ne and bottleneck intensity and duration. Several studies have tested for a bottleneck just after introduction by simulating a reduction in Ne during few generations followed by a larger stable Ne (Facon, Hufbauer, et al., 2011; Kerdelhué et al., 2014; Sherpa, Blum, Capblancq, et al., 2019). However, this approach has its own limitations because it requires discrete times for contraction/expansion or admixture events, and the proper design of prior distributions for all parameters (the coalescent Ne varies with the expected time of coalescence).

Several coalescent methods have been developed to reconstruct continuous fluctuations in Ne over time (Beichman et al., 2018). These methods rely on the use of summary statistics, such as the site frequency spectrum (SFS), the linkage disequilibrium (LD) at different physical distances, or both (Table 2; Appendix S1). Particularly useful for timing deep demographic events for a limited number of samples (Mather et al., 2020), they are probably not appropriate for studying invasion timescales. Most of the assumptions of these methods (i.e., panmixy, no migration) are violated in the case of invasive populations because recent history often comprises inbreeding or multiple introductions. Analyzing genetically homogeneous groups may reduce the confounding effect of population structure in demographic inferences (Chikhi et al., 2010) but recent migration (multiple introductions) can create artifacts of population contraction. Most of these methods require a high‐quality reference genome assembly but RADseq datasets seem to contain the coalescent information to reconstruct introduction histories (Liu & Hansen, 2017; Sherpa et al., 2018).

Effective population size is not a direct estimate of population invasiveness but can be used to evaluate the relationship between introduction modalities and the result of an introduction based on correlative patterns. Do highly diverse introduced populations tend to initiate more often secondary introductions (number of events) and/or spread farther or faster (geographic distance or occupied area) than low‐diversity ones? Concerning the evolution of fitness in introduced populations, the genetic load can be estimated from genomic data (Henn et al., 2015), for example by screening the accumulation of deleterious mutations at genes involved in reproductive traits. Long‐established invasive populations are expected to accumulate new deleterious mutations masking initial genetic load purging in natural populations, except if a key temporal sampling can be performed in order to compare the genetic composition of the intercepted migrants arriving in the new range and the established population after several generations.

### 3.2 Introduction modalities based on phenotypic experiments

Population genetics studies revealed that propagule pressure might be common in insect invasions (Lockwood et al., 2005) but distinguishing large founding size and diversity from propagule number is impossible from molecular data. Admixture is often observed in invasive species that have undergone extreme range expansions (Fraimout et al., 2017; Lesieur et al., 2019; Sherpa, Blum, Capblancq, et al., 2019) supporting the heterosis hypothesis but only quantitative genetics studies can effectively address its role in the invasion success. Finally, several studies found genetic diversity loss between introduced populations and their source (Garnas et al., 2016; Uller & Leimu, 2011), which did not necessarily prevent some introductions to successfully establish (Arca et al., 2015; Puillandre et al., 2008; Schmid‐Hempel et al., 2007; Zayed et al., 2007). Based on the relationship between genetic diversity and the evolution of genetic load and inbreeding depression (Kirkpatrick & Jarne, 2000), one could explain the latter pattern by inbreeding‐by‐environment interactions (Schrieber & Lachmuth, 2017) or by deleterious mutation purging (Glémin, 2003) in the absence of environmental filtering. However, assessing inbreeding depression in introduced populations requires measuring individual fitness traits in addition to inbreeding coefficients (Hoffman et al., 2014; Kardos et al., 2016).

Quantitative genetic experiments have been useful to give a theoretical understanding of the role of propagule pressure, admixture events, and the purging of genetic load in the invasion success. However, theoretical results are difficult to apply to invasive populations because (i) only successfully established populations are observed, and (ii) demographic parameters such as population growth or bottleneck intensity are hardly measurable in the field. For example, quantifying population growth in natural populations would require tedious MRR experiments as soon as new arrivals have been detected and over several generations and then compare the outcome of each detection with the reconstructed invasion history (number of sources and founding diversity). Experimental introductions manipulating the genetics and demography simultaneously have shown that establishment success (i.e., successful reproduction and population growth) increases with the density and/or diversity of founders but depends on the species (Szűcs et al., 2014 and references therein). Using the knowledge provided by both colonization routes and demographic inferences to design phenotypic experiments makes it possible to test the impact of observed demographic changes for one specific introduction event while comparing relevant genetic units (introduced and source populations). For example, controlled crosses in *H*.* axyridis* evaluated the respective roles of admixture and inbreeding in reducing the genetic load in invasive populations (Box 2, Figure 2). Modeling the evolution of allele frequencies allows predicting which combination of demographic parameters during introduction may favor the purging of highly deleterious alleles but not to predict the outcome of an introduction as the evolution of genetic load (accumulation of slightly deleterious or highly recessive alleles in the genome) through drift is a stochastic process.

## 4 THE EXTENT AND OUTCOME OF ENVIRONMENTAL FILTERING

Understanding the genetic basis of traits involved in the rapid adaptation of introduced populations is a major objective in invasion genetics. General guidelines integrating several steps highlight the benefit of using complementary approaches and various types of data (Bertelsmeier & Keller, 2018; Hufbauer et al., 2012; Rey et al., 2012). The first step is to reconstruct the colonization routes, because any adaptive genetic variant within the introduced range can simply reflect the preexisting variation within the source range. The second step is to demonstrate that established populations faced new selective pressures during the colonization process. The classically proposed third step is to perform phenotypic analyses (Bertelesmeier & Keller, 2018; Rey et al., 2012). In contrast, we recommend to first search for genomic signatures of selection and then perform fitness comparison of the divergent genotypes (Swaegers et al., 2015).

### 4.1 Environmental filtering based on ecological niches

**FIGURE 3:**
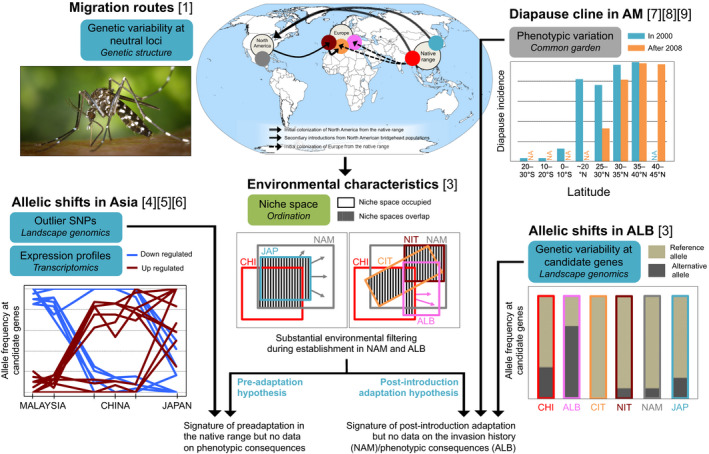
Types of information needed to test the preadaptation and post‐introduction adaptation hypotheses using studies in *A*.* albopictus*. Blue: genetic variability, green: environmental and occurrence data, orange: historical data, gray: phenotypic data. Photo of *A*.* albopictus* from https://commons.wikimedia.org/wiki/ File:CDC‐Gathany‐Aedes‐albopictus‐1.jpg (CC0, James Gathany). Figures adapted from cited literature

Before questioning the role of adaptive evolution in promoting invasion, it is important to quantify the level of environmental constraints faced during introduction. Two main approaches can be used to compare niches between invaded and source ranges: ordination‐based methods and SDM (Broennimann et al., 2012). Both approaches have weakness and strengths, and several metrics in each approach allow evaluating niche changes (Guisan et al., 2014). Most comparisons of climatic niches have been performed between global invasive and native ranges (Guisan et al., 2014). However, because introduced populations can originate from bridgehead invasive populations, this step must be conducted between invaded and source (i.e., not native) ranges. Based on colonization routes and SDM overlaps between climatic niches occupied by the invaded populations and their source(s), niche conservatism in climatic tolerance was demonstrated in the ant *Wasmannia auropunctata* (Rey et al., 2012) and *A*.* albopictus* (Sherpa, Guéguen, et al., 2019). Niche comparisons should explore both ordination and SDM techniques as they can provide contrasting results (Broennimann et al., 2012). For instance, SDM predicted a high stability of the niche during the European invasion of *A*.* albopictus* whereas PCA‐based comparison revealed substantial changes (Box 3, Figure 3).

BOX 3 **The invasion of temperate regions by *Aedes albopictus***

**Routes of colonization and niche comparisons**

From its native Asian range, the tiger mosquito *A*.* albopictus* has been accidentally introduced in all continents but Antarctica in the last decades. The reconstruction of colonization routes demonstrated a bridgehead scenario, involving the colonization of NAM from China (CHI) and Japan (JAP) (admixture event 1) that served as the source for the initial colonization of North Italy (NIT), and independent introductions from CHI in Albania (ALB, single source) and Central Italy (CIT) (admixture event 2) (Sherpa, Blum, Capblancq, et al., 2019). Most SDM studies compared invaded range niches to those of the entire native range without taking the origin of populations into account, for example (Cunze et al., 2018). Based on colonization routes and using both SDM and ordination methods, (Sherpa, Guéguen, et al., 2019) demonstrated an overall good overlap between the niche of introduced and source populations (Figure 3).

**Genomic signatures of cold adaptation**

The success of *A*.* albopictus* invading temperate regions has been attributed to the photoperiodic diapause response of eggs allowing the species to survive winters in cold environments. Recent work using NGS technologies (RNA‐seq (Huang et al., 2015; Poelchau et al., 2013) and RAD‐seq (Sherpa et al., 2019)) has provided extensive insight into the genetic basis of this response. Transcriptome analyses revealed that thousands of genes are differentially expressed between mosquitoes reared under diapause and non‐diapause inducing photoperiods (Huang et al., 2015; Poelchau et al., 2013). Landscape genomic analyses of population differentiation between three native ecogeographic regions found outlier SNPs located in or near candidate transcripts for diapause (Sherpa, Blum, & Després, 2019) (Figure 3). These results suggest that the key ‘genetic toolkit’ for invading temperate regions was already present in northern latitude of the native range.

**Phenotypic characteristics along temperate gradients**

Diapausing populations survive better than non‐diapausing populations to cold exposure, with similar response in NAM and JAP (Lounibos et al., 2003). Temporal and spatial comparisons of this cold‐adapted phenotype along the NAM climatic gradient suggest rapid local adaptation after introduction (Lounibos et al., 2003; Medley et al., 2019; Urbanski et al., 2012) (Figure 3). However, the lack of knowledge on egg survival along the large native range and on the history of introductions in NAM prevents deciphering between local adaptation and preadaptation. Future research should focus on the possible establishment of different founding populations in NAM with different diapause responses, and the genetic variation at diapause genes in invasive populations. For instance, genetic studies found genetic differences between southern and northern NAM populations (Kotsakiozi et al., 2017) and multiple sources in southeast NAM (Sherpa, Blum, Capblancq, et al., 2019).

In addition to climate, land‐use data and human footprint indexes should be considered because adaptations to human‐modified habitats within the native range might further enhance the likelihood of establishment into a novel range (Hufbauer et al., 2012). For example, *W*.* auropunctata* invasive and native populations living in human‐modified habitats display a larger thermotolerance than native populations living in natural habitats (Foucaud et al., 2013). Furthermore, populations in areas frequented by humans are more likely to be transported. In *A*.* albopictus*, invasive populations only breed in artificial containers, which initially results from an ecological shift from natural (tree hole) to human‐modified (used tires, cemetery vases) environments within its native range, and artificial containers are the mode of its introduction worldwide. Because the realized niche also includes biotic interactions, the absence of parallels between climatic distribution in invaded and source ranges may reflect competitive interactions setting each range limits (Keane & Crawley, 2002; Stachowicz & Tilman, 2005). The use of biotic predictor variables in invasive SDM remains scarce (Berzitis et al., 2014) probably because it requires knowledge on the suspected interactions. Co‐occurrence data (e.g., joint SDM; Norberg et al., 2019) and molecular methods (e.g., DNA barcoding; Viard & Comtet, 2015) exist to detect potential biotic interactions.

Niche comparisons help defining the temporal and spatial scales for detecting adaptive shifts in invasion landscape genomic studies: (i) before introduction by studying adaptive variation within the source range (preadaptation, e.g., Sherpa, Blum, & Després, 2019), (ii) during introduction by comparing the intercepted migrants arriving in the new range and its source (selection during transportation, e.g., Briski et al., 2018), (iii) during establishment by comparing the introduced populations at primary sites of detection and their source(s) (postintroduction adaptation, e.g., Sherpa, Guéguen, et al., 2019), and/or (iv) during expansion by comparing range core and range edge populations within the invaded range (spatial sorting, e.g., Swaegers et al., 2015).

### 4.2 Environmental filtering based on landscape genomics

Landscape genomics has become a powerful method to detect the genetic variants under selection out of a large set of molecular markers without measuring phenotypes (i.e., genome scans outliers, Beaumont & Balding, 2004). A large number of methods have been developed, including population‐based approaches and individual‐based ordination methods (Hoban et al., 2016; Li et al., 2017 for reviews; Table 2). The controversial topics in landscape genomic studies include the sampling strategy, the method, and the test for significance (Li et al., 2017; Manel et al., 2012; Appendix S1). Methods using only genetic information are based on between‐population differentiation and do not link the outlier loci to specific environmental variables. They can be useful when the selective pressures are evident or known (Sherpa, Blum, & Després, 2019) or when populations have the same neutral genetic background (Cattel et al., 2020). However, sampling many individuals per population is required to accurately estimate allele frequencies. Genome–environment association tests are correlative approaches explicitly integrating genetic information and environmental variation. Because these methods assume that genes involved in local adaptation present shifts along environmental gradients, sampling multiple populations is required to accurately estimate allele frequencies at each position of the environmental gradient (Sherpa, Guéguen, et al., 2019). The complex demographic history of invasive populations, such as stochastic loss of alleles through drift and gain of alleles after multiple introductions, can lead to the artifactual detection of selection signals (i.e., false positives). Several statistical approaches nevertheless account for the confounding effect of population structure and minimize the false discovery rate (Appendix S1).

The ultimate aim of landscape genomic studies is the indirect validation of candidate loci through the elucidation of gene function. Candidate loci are more likely to be physically linked with the selected loci than to be the causal mutation itself (Nordborg & Tavare, 2002), but represent a first step in localizing the genes involved in adaptation. Most conclusive studies include molecular markers in both anonymous and candidate regions (i.e., genes coding for traits known to be involved in local adaptation) together with functional validation. In *A*.* albopictus*, transcriptome analyses combined with physiological experiments provided relevant information to identify candidate regions for cold adaptation among outlier SNPs (Box 3, Figure 3). Most of invasive species are nonmodel species and the detected candidate loci do not have functional annotation. A recent genome‐wide association study in *D*.* suzukii* identified several genes that could be involved in the invasion of NAM and Europe (Olazcuaga et al., 2020) based on previously identified colonization routes (Fraimout et al., 2017). However, the lack of functional characterization of the genome and of the environmental characteristics of the invaded and source ranges makes the interpretation of the results challenging (Olazcuaga et al., 2020).

In invasion studies, admixture/hybridization events are commonly characterized using global ancestry coefficients (population structure) or using estimation of genomic admixture rates as a component of demographic history (colonization routes). However, a large number of tools, based on genetic differentiation, local ancestry inference, or phylogenetic relationships, have been specifically developed to detect recent introgression and to determine the population of origin of introgressed fragments (Table 2; Appendix S1). Introgression of genetic variation from a donor species into a recipient can provide adaptive advantages, and whole‐genome data provided information about the role of adaptive introgression in expansion success. For instance, in the honey bee *Apis mellifera* expanding from Africa to Europe and SAM, European‐derived alleles and positive signatures of selection preferentially located in coding regions were detected in the genome of the most invasive SAM lineage (Zayed & Whitfield, 2008). Adaptive introgression has also been shown among *Anopheles gambiae* species complex with evidence for stress‐related (desiccation, immunity) and insecticide resistance alleles being preferentially transferred from a species to another (Clarkson et al., 2014; Fouet et al., 2012; Mendes et al., 2010).

### 4.3 Environmental filtering based on phenotypic experiments

The functional relevance of genes identified using landscape genomics remains unknown until the observed genetic changes have been linked to phenotypic changes of the selected trait. Establishing this causative link represents one of the greatest challenges in invasion genetics. First, it requires experimental settings in order to disentangle phenotypic plasticity from adaptive evolution in natural populations. The role of phenotypic plasticity in biological invasions has been reviewed elsewhere (Renault et al., 2018) and is beyond the scope of our review. Demonstrating that the phenotypic variation measured between populations reflects their genetic difference can be assessed by common garden experiments. These experiments allow identifying phenotype–environment clines reflecting local adaptation (Box 3, Figure 3). Only reciprocal transplant experiments provide evidence for the selective advantage of the focal phenotype but this type of experiments is rife with ethical problems in the context of biological invasions. Indeed, it can enhance gene flow between two areas within the introduced range, re‐introduce genetic variation, or introduce invasive populations in not occupied areas, which can all have ecological and evolutionary consequences on local invasive populations (promoting invasiveness) and ecosystems (promoting new introductions). To reduce these risks, transplanted populations could be removed before they complete their reproductive cycle in plants, or be reared in field cages to avoid uncontrolled dispersal.

Second, neutral and adaptive phenotypic variance in invasive populations is impacted by both the origin of populations and introduction modalities. Comparing an introduced population to its source and characterizing its demographic history is thus needed to accurately detect phenotypic changes in response to natural selection in the invaded area (Keller & Taylor, 2008). The differentiation at quantitative trait loci (QTL) (*F*_STQ_) as compared to neutral genetic differentiation (*F*_ST_) and phenotypic differentiation (*Q*_ST_) has been largely used to detect the loci targeted by selection (Le Corre & Kremer, 2012; Leinonen et al., 2013) and several quantitative trait detection methods now include population structure to account for shared neutral trait divergence among populations (de Villemereuil et al., 2020 and references therein).

Third, adaptive phenotypes such as stress and thermal tolerance as well as dispersal and reproductive potential are presumably controlled by a large number of loci. In this multigenic case, local adaptation is reached by increased covariance of allelic effects rather than via allele frequency changes (Le Corre & Kremer, 2012). The recent completion of whole‐genome sequencing for many organisms and the development of association mapping methods will improve the understanding of the evolution of complex invasive phenotypes (Savolainen et al., 2013; Stinchcombe & Hoekstra, 2008). For example, a combination of QTL mapping and genome‐wide association analysis allowed to dissect the genetic architecture of a complex adaptive quantitative phenotype in *Aedes* mosquitoes, the resistance level to *Bacillus thuringiensis israelensis* toxins used in biological control, and to identify strong candidate genes (Bonin et al., 2015).

## 5 RECONSTRUCTION OF THE EXPANSION HISTORY

### 5.1 Forecasting expansion based on ecological niches

**FIGURE 4:**
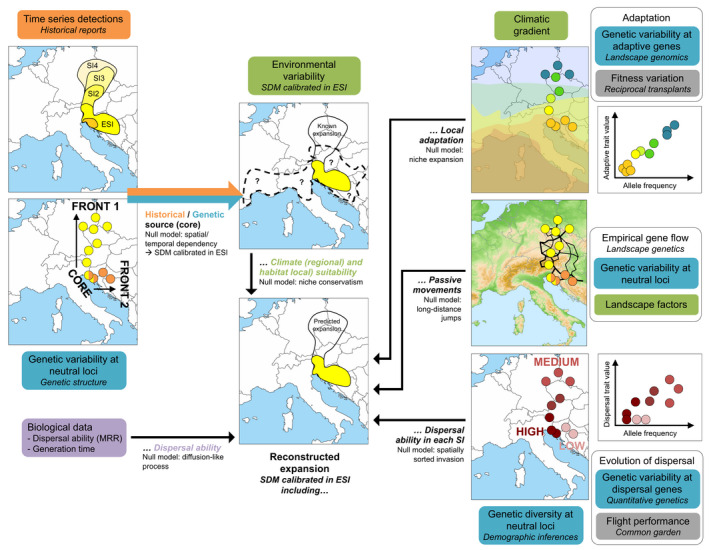
Types of information needed to assess the spread dynamics of invasive species and to reconstruct the expansion history. Blue: genetic variability, orange: dated introductions, green: observational and environmental data, violet: biological data, gray: phenotypic data. For the illustration, the studied invasion process is the northward expansion (CORE vs. FRONT 1). SI: stage of invasion; ESI: early stage of invasion

Species distribution models are often used to predict future invasive species distribution under current climate (Fournier et al., 2019; Peterson & Vieglais, 2001) or climate change (Bellard et al., 2013) but rarely to reconstruct the expansion history. For instance, by decomposing time‐series detections into two stages of invasion, Barbet‐Massin et al. (2018) demonstrated that the recent expansion (occurrences from the late stage of invasion) of the Asian hornet *Vespa velutina* in France was well predicted by SDM calibrated using the occurrences from the earlier stage of invasion (ESI). This approach requires identifying the core and edge populations, which is difficult in the absence of dated introductions but genetic diversity gradient along the expansion axis can inform about the location of ESI (Figure 4).

Integrating global (calibrated in the source area) and regional (calibrated in the invaded area) models and multiple environmental predictors (climate and land use) can significantly improve invasive range predictions especially for ongoing expansion violating the equilibrium assumption of SDM (Fournier et al., 2017; Gallien et al., 2010, 2012). However, the spread dynamics of invasive species is also affected by a large number of processes including species biology, population demography, and local adaptation not accounted for so far in SDMs (Peterson et al., 2019), as well as human‐aided long‐distance dispersal. After identifying (i) the most likely source (core) of edge populations (colonization routes) and (ii) the spread dynamics: Ne (demographic inferences), dispersal abilities from direct (biological data, experiments) or indirect approaches (landscape genetics), and local adaptation (landscape genomics), SDM could be used to (iii) retrace the expansion history from ESI by incorporating one or multiple parameters of the spread dynamics, and (iv) compare the predicted distributions calibrated in ESI to the observed expansion (Figure 4).

### 5.2 Forecasting expansion based on dispersal abilities

Dispersal can result from the combination of passive human‐aided dispersal, and active local diffusion (i.e., stratified dispersal) (Wilson et al., 2009). The contribution of natural dispersal to expansion received little attention so far in invasion studies because active movements are thought to be of minor importance in the face of human‐aided long‐distance dispersal for invasive species having low dispersal capabilities. Understanding the role of natural dispersal at the local scale can be assessed by incorporating information on the species’ dispersal ability in SDMs (Engler et al., 2012). Dispersal abilities estimated from MRR provide particularly relevant data to model potential local expansion of invasive species (Marcantonio et al., 2019). For example, MRR data were used to build a backward dispersal model in a recently invaded urban area by *A*.* albopictus* (Sherpa et al., 2020). Year‐to‐year comparisons of occupancy data, taking into account the maximum possible number of generations from occurrence dates given generation time, fixed dispersal abilities, and habitat suitability, allowed to estimate the role of natural dispersal in promoting the expansion of populations. Such dispersal model assumes that the occurrence dataset represents the distribution of the species at each temporal step and that the dispersal ability remains constant at each step of the invasion. Because natural dispersal acts at the local scale, the dispersal abilities should be included in local land use‐based SDM but not in global climate‐based models in which migration is mainly driven by passive movements. Combined with landscape genetics, all these observational, experimental, and model‐inferred information may help to understand how post‐introduction dispersal and landscape features shape spatial patterns of genetic variation.

### 5.3 Forecasting expansion based on empirical gene flow

Landscape genetics aims at determining the local factors shaping population connectivity (Manel et al., 2003) and should provide a better understanding of the role of dispersal in the expansion process. Based on the causal relationship between dispersal and gene flow, the genetic and geographic distances between populations have been used to quantify the effective dispersal across populations (i.e., isolation by distance, isolation by barriers). Recent approaches aim at quantifying the distance between populations according to the landscape features (i.e., isolation by resistance; Table 2; Appendix S1).

At the regional scale, the topography of landscape features also shapes human activities, thereby impacting indirectly population connectivity (i.e., long‐distance jumps), as demonstrated by landscape genetics surveys in *A*.* albopictus* showing that long‐distance dispersal is facilitated along roads (Medley et al., 2015; Sherpa et al., 2020). SDMs could be useful to understand to which extent human activities contribute to invasion speed (population persistence at range edges). The abundance of artificial habitats created by human activities (local land use‐based models) and passive movements (anthropogenic networks) can promote the expansion of invasive populations in unsuitable climatic areas (regional climate‐based models) (Figure 4). For instance, greenhouses provided suitable microhabitats for the expansion of *Thrips palmi* in Northern China where the species can probably not overwinter outdoors (Cao et al., 2019).

At the local scale, the genetic structure of populations can partly reflect natural dispersal, which can be influenced by geographic distance, by environmental barriers, or by resistance of land‐cover features. For example, testing land‐cover features as a barrier or corridor for gene flow between populations, Sherpa et al. (2020) demonstrated that populations in diffuse urban areas tend to disperse less while roads facilitate long‐distance dispersal. Studying isolation by resistance requires a precise knowledge on the expansion history. Indeed, genetic structuring among invasive populations can result from independent bottlenecks in geographically isolated multiple introductions or a unique introduction and further differentiation due to drift and reduced gene flow. When biological data are not available, such as species dispersal ability, empirical gene flow could be used instead for constraining the expansion routes with landscape boundaries (e.g., mountain chains).

### 5.4 Forecasting expansion based on evolutionary dynamics

One of the main traits promoting invasiveness is dispersal ability. The selection of traits increasing dispersal ability during range expansion (i.e., spatial sorting, Phillips & Perkins, 2019) has been experimentally demonstrated in *Callosobruchus maculatus* and *Tribolium castaneum* (Ochocki & Miller, 2017; Weiss‐Lehman et al., 2017). These two common garden experiments compared the dispersal distances between the leading edges of spatially sorted replicate invasions and a control that was built by redistributing individuals randomly to prevent the carrying of alleles for high dispersal. In both studies, spatially sorted invasions spread farther than shuffled invasions on average. Demonstrating increased dispersal rates as a result of spatial selection in natural populations requires comparing populations that are part of a single spread (same genetic background) and along neutral genetic diversity gradients. Indeed, this process is only observed in low‐density populations with assortative mating, whereas high‐density populations (Weiss‐Lehman et al., 2017) and outbred hybrid populations (Wagner et al., 2017) have in any case higher dispersal capacities. Yet, such density‐dependent adaptive pattern was observed in natural expanding populations of *H*.* axyridis* (Lombaert, Estoup, et al., 2014) and was correlated with allele frequency changes at one locus associated with flight endurance in *Coenagrion scitulum* (Swaegers et al., 2015).

Spatial bottlenecks can compromise local adaptation at range edges due to expansion load but gene flow between range and core can slow range expansion due to the introduction of maladapted alleles that counter local adaptation (Verhoeven et al., 2011). Nonetheless, some studies investigating adaptive changes during range expansion of invasive species found significant shifts along climatic gradient, suggesting a selection for cold‐resistant phenotypes in range edge regions with lower temperatures (Medley et al., 2019; Swaegers et al., 2015; Urbanski et al., 2012). A new generation of SDMs that explicitly integrate fitness‐related trait variation into habitat suitability prediction is currently developing (Benito Garzón et al., 2019; Peterson et al., 2019). This approach is so far restricted to forest trees in the face of climate change but might also benefit invasive species range expansion predictions. More complex models could incorporate (i) the probability of population to persist and expand (genetic diversity), (ii) the potential for dispersal traits to evolve under selection operating through space, and/or (iii) the adaptation of populations along environmental gradients during range expansion (Figure 4).

## 6 CONCLUSIONS AND PERSPECTIVES

The demo‐genetic and evolutionary processes at play during an invasion have been extensively reviewed (Table S1). Yet, the key determinants of successful invasions remain largely unclear because of the numerous factors interacting. Indeed, the demographical dynamics and complex genetic structure of invasive species makes particularly challenging the study of their evolutionary responses to new environments. We advocate for a more integrative invasion biology science that takes advantage of multiple types of information (historical, genetic, environmental, phenotypic) and of up‐to‐date analytical tools. The integrative approach requires effective collaborative academic and nonacademic multidisciplinary networks in order to (i) improve the quality and complementarity of datasets and (ii) develop more integrative analytical methods.

Future progress in invasion science relies on the quality of time‐series occurrence datasets and sample collections, which need to be accessible (e.g., research data publications or archiving, monitoring, and management programs results). Historical data are still under‐represented in invasion studies because early detection of introductions and careful monitoring of further expansion are challenging and costly. However, SDM might not be conclusive if based on a limited number of occurrences. Participative citizen science projects represent an interesting alternative to monitoring structures, and will potentially provide huge occurrence datasets, providing the invasive species is easily identified, fueling distribution and evolutionary models (Ryan et al., 2019). Remote sensing (satellites, drones) observations of spatiotemporal damages as a proxy for forest and/or crop pests presence offers promising perspectives to improve time‐series occurrence datasets, while the use of environmental DNA to track invasive species is already widely used (Baudry et al., 2020; Young et al., 2021). Public databases make readily accessible worldwide climate data at high resolution while access to land‐cover information is more region‐dependent, and not always available at the spatiotemporal scale required. In addition to land‐cover information, detailed local information about other species possibly interacting with the target species (e.g., host‐plant, predators, competitors) should ideally complete the environmental predictors to be used for habitat suitability evaluation, but SDMs integrating biotic components as predictors are still in their infancy (Norberg et al., 2019). Public genomic databases give access to large and ever‐increasing amount of genomic and transcriptomic information and the issue of nonmodel species lacking annotated reference genome will presumably be soon overcome.

More integrative analytical methods that take into account simultaneously environmental, ecological, phenotypic, and genomic datasets are still to be developed. While integrating ecological models in evolutionary studies is helpful to delineate the neutral and adaptive processes shaping diversity patterns at different spatial scales, a better use of evolutionary models (adaptive potential at range edges) and of changes overtime in human activities in ecological studies would allow to go a step further in our capacity to predict future invasive range expansions. Developing new methods explicitly incorporating different types of data rather than performing separately different types of analyses represents the future challenge in invasion science.

## ACKNOWLEDGEMENTS

We thank Nicolas Bierne and three anonymous reviewers for helpful comments on a previous version of the manuscript.

## CONFLICT OF INTEREST

The authors declare that they have no conflict of interest.

## Supporting information

Appendix S1

Table S1

## Data Availability

Data sharing not applicable to this article as no datasets were generated or analyzed during the current study.
