# Supplementary material for: The evolutionary dynamics of biological invasions: A multi‐approach perspective
Source: Evol Appl. 2021 Mar 30;14(6):1463–84. doi: 10.1111/eva.13215 (PMC8210789; doi:10.1111/eva.13215)
Supplement: Supplementary file 1 — Appendix S1 [file EVA-14-1463-s002.docx]

**Appendix S1**

**Methods used for examining population processes in the context of biological invasions based on genetic data**

**1. Genetic structure and colonization routes**

The source of first introduction has been classically deduced from measures of genetic differentiation among populations, such as *F*_ST_. However, population-based approaches require defining populations (groups of individuals exchanging genes: island models), which is problematic in the case of dynamical processes such as biological invasions. Individual-based assignment methods provided considerable advances (Rannala & Mountain, 1997; Cornuet et al., 1999; see main text Table 2) and have been largely used to identify recent migration events. For example, using GeneClass2 (Piry et al., 2004), Ciosi et al. (2008) revealed several independent introductions of the western corn rootworm in Europe from its North American native range. Other clustering methods, such as STRUCTURE (Pritchard et al., 2000), are commonly used to determine the origin of the invasive population: its clustering with one of the potential source populations is further interpreted in the light of geography and natural history (e.g. Bittner et al., 2017). However, these methods do not account for demographic stochasticity (i.e. bottleneck effects) and multiple introductions, which is pervasive especially at the first steps of an invasion (Estoup & Guillemaud, 2010).

Much more model-flexible approaches are now available to determine source populations while allowing for complex demographic history (Estoup & Guillemaud, 2010). Unlike classical population models (Wright-Fisher forward models), coalescent models reconstruct gene genealogies back in time (backward models). Gene genealogies can be computed given different demographic scenarios and compared by their fit to the observed dataset (summary statistics) in the approximate Bayesian computation (ABC) framework. Coalescent modelling coupled with ABC, such as implemented in DIYABC (Cornuet et al., 2008), allows testing for different introduction scenarios, involving population split, size changes (bottleneck, expansion) and admixture events, and their timing. This framework is the most commonly used for reconstructing invasion routes nowadays but requires a rigorous design of alternative scenario topologies and of prior parameters. ABC methods first require to pool individuals into samples between which the evolutionary relationships are tested, and pooling highly differentiated samples can lead to erroneous conclusions regarding the origin of introduced populations (Lombaert et al., 2014). It is therefore advisable to use classical population-based methods to define the genetic clusters on which further historical demographic inference can be done (see main text Table 2; Excoffier & Heckel, 2006 for a review). Given the very large number of plausible scenarios for introduction and the impact of sampling intensity on the results, conducting a step-by-step analysis guided by genetic structure or including an unsampled ‘ghost population’ is particularly useful (see main text).

A thorough evaluation of the quality of the models can give confidence in the results (Cornuet et al., 2010; Robert et al., 2011). This includes (i) checking that the scenarios compared have distinct posterior distributions and that the best scenario includes observed data in its posterior distribution space and (ii) evaluating the confidence in scenario choice by simulating datasets under each scenario and measuring the proportion simulated under the best scenario that were assigned to other scenarios (type I error) and the proportion simulated under other scenarios that were assigned to the best scenario (type II error) (Cornuet et al., 2010). An alternative solution is to randomly sample individuals in each genetic cluster and run the analysis several times on different subsets (Lombaert et al., 2014; Guillemaud et al., 2015).

**2. Demographic inferences**

Since the 1970s, a large number of methods have been developed to reconstruct the demographic history of populations from genetic data (Salmona et al., 2017). In invasion genetics, the demographic parameters of introduced populations have been classically measured by population genetic diversity indices. Several tests have also been developed to detect deviation from constant population size over-time (null hypothesis: mutation-drift equilibrium, no migration) for diploid multilocus genotypes and single locus DNA sequences, such as implemented in BOTTLENECK (Piry et al., 1999) and DnaSP (Rozas et al., 2003) respectively (see main text Table 2). However, the reliability of these tests to detect demographic changes at the time-scale at which biological invasions occur has been largely questioned (Peery et al., 2012; Fitzpatrick et al., 2012). Indeed, deviation from mutation-drift equilibrium appears after several generations of reduced population size and subsequent immigration can make bottlenecks undetectable.

Significant progress has been made with the development of the coalescent theory (Salmona et al., 2017). The ABC methods allow inferring population demographic parameters, including effective population size and bottleneck intensity. Several studies have tested for a reduction in effective population size during few generations followed by a larger stable effective population size by simulating a bottleneck period just after introduction (e.g. Facon et al., 2011; Kerdelhué et al., 2014; Sherpa et al., 2019a). However, this approach has its own limitations because it requires discrete times for contraction/expansion or admixture events and the proper design of prior distributions for all parameters, which represents the main challenge of this approach.

Recently, several methods have been developed to reconstruct continuous fluctuations in effective population size over time (see main text Table 2; Beichman et al., 2018). Although most require a high-quality reference genome assembly and linkage map, such as PopSizeABC (Boitard et al., 2016) and sequentially markovian coalescent series of model (Li & Durbin, 2011; Schiffels & Durbin, 2014; Terhorst et al., 2017), those using unphased data, such as the stairway plot method (Liu & Fu, 2015) represent a good alternative. This method suggested that not only past (e.g. Pleistocene climatic fluctuations within the native range of *A. albopictus*, Sherpa et al., 2019b) but also recent (e.g. introduction history of *A. aegypti* in the Caribbean, Sherpa et al., 2018) changes in effective populations could be retrieved from reduced genome-wide representations of the genome. However, these methods require to fix the spontaneous mutation rate, which influence the inferred parameters. Accurate estimations using mutation-accumulation lines experiments and whole genome sequencing are thus needed. Furthermore, most of the assumptions (i.e. panmixy, no migration) are violated in the case of invasive populations, which recent history often comprises inbreeding or multiple introductions. Analyzing genetically homogeneous groups may reduce the confounding effect of genetic structure in demographic history inferences (Städler et al., 2009; Chikhi et al., 2010).

**3. Genomic signatures of local adaptation**

Methods using only genetic information to detect genomic regions putatively under selection include population-based approaches and individual-based ordination methods (see main text Table 2). Population-based methods (reviewed in Hohenlohe et al., 2010), such as BayeScan (Foll & Gaggiotti, 2008) and OutFLANK (Whitlock & Lotterhos, 2015), use the between-population differentiation (*F*_ST_) at each single locus to identify genomic regions more differentiated than the average differentiation across the genome (i.e. outlier loci). Individual-based ordination methods, such as PCAdapt (Luu et al., 2017), have been developed to overcome the difficulty to group individuals into populations, and can handle admixed individuals. However, these methods fail to detect outliers when the overall differentiation at neutral loci is high due to divergent independent evolution. Furthermore, they do not link the outlier loci to specific environmental variables.

Methods that explicitly integrate genetic information and environmental variation also include population-based approaches and individual-based ordination methods (see main text Table 2). Genome-environment association methods (reviewed in Rellstab et al., 2015), such as LFMM in R (Frichot et al., 2013), BayPass (Gautier, 2015) and BayeScEnv (De Villemereuil & Gaggiotti, 2015), are linear mixed models that identify correlation between genetic and environmental variation, but each environmental variable is tested separately. Individual-based ordination methods, such as rdadapt in R (Capblancq et al., 2018), provide a useful alternative when the main selective pressure is unknown, by allowing to test simultaneously for association with several environmental variables. This multifactorial approach also estimates the proportion of the genome that is constrained by environment. Because these methods assume that genes involved in local adaptation present shifts along environmental gradients, populations should be continuously sampled along the gradient.

The genomic sampling effort can significantly impact the performance of these methods in identifying SNPs under selection (Arhens et al., 2018) but their main drawback stems on the complex demographic histories of invasive populations: founder effects, loss of alleles through drift, gain of alleles after multiple introductions, and stochastic fixation of alleles in expanding wave fronts (i.e. allele surfing), can all lead to the artefactual detection of selection signals (i.e. false positives). Among the listed methods, BayeScan, BayeScEnv, and BayPass are based on the same *F*-model that explicitly accounts for population structure by using population-specific *F*_ST_ measuring the degree of genetic drift in each population (Foll & Gaggiotti, 2008; De Villemereuil & Gaggiotti, 2015). In LFMM, the genetic structure of populations is accounted by the confounders (i.e. latent factors) corresponding to the number of genetic clusters observed among populations (Frichot et al., 2013). rdadapt and generalized dissimilarity modelling (GDM; Fitzpatrick & Keller, 2015; Manion et al., 2017) can take into account the spatial autocorrelation between geography and environment as well as the confounding effect of population structure. This can be done simply in rdapdat by adding a covariate in the model, removing the effects of genetic structure (admixture coefficients from any clustering analysis) or geography (sampling coordinates). GDM can use a random subsample of molecular markers to build a null model (i.e. reference), which tests the probability of detecting an outlier locus by chance (Fitzpatrick & Keller, 2015), as well as the effect of isolation by distance to test whether candidate loci are more constrained by environment than geography (e.g. Sherpa et al., 2019c). The false discovery rate can be further reduced using a *Q*-value framework and the outliers that are detected by several different methods can be retained as good candidates (de Villemereuil et al., 2014).

**4. Admixture/Hybridization and adaptive introgression**

Characterizing admixture and hybridization events (time, proportion) between divergent lineages and introgression of genetic material from a species to another is possible through statistical tests on genetic variation without necessarily requiring whole genome data (e.g. Geneva et al., 2015; Racimo et al., 2015; Rosenzweig et al., 2016). A common approach to detect introgressed fragments is to test for an excess of shared derived variants in the donor and recipient populations using the D or ABBA-ABBA test statistic (Durand et al., 2011), but extreme D values occur disproportionately in genomic regions with lower diversity (Martin et al., 2015). Patterson et al. (2012) formalized statistical approaches to estimate admixture based on allele frequencies across multiple populations, namely the f3 and f4 statistics. More powerful statistics have been developed such as the fD (Pfeifer & Kapan, 2019) or BDF (Martin et al., 2015) statistics, but they cannot be used to identify introgressed regions between sister species. A large number of programs are available for calculating these statistics, such as AdmixTools (Patterson et al., 2012) and Comp-D (Mussmann et al., 2019).

Several methods have been developed to assign genomic regions to donor populations based on patterns of allele or haplotype sharing (i.e. local ancestry inference LAI, Geza et al., 2018). Most LAI software packages require phased haplotypic data (e.g., HapMix, Price et al., 2009; RFMix, Maples et al., 2013; Loter, Dias-Alves et al., 2018) but some do not (e.g. WINPOP, Paşaniuc et al., 2009; ELAI, Yang et al., 2013) and some can even handle SNP data such as PCAdmix (Brisbin et al., 2012), Bayesian STRUCTURE analysis (Pritchard et al., 2000) and TreeMix topologies (Pickrell & Pritchard, 2012). These methods strongly vary in the biological/statistical parameters used (e.g., linkage disequilibrium, mutation and recombination rates, number of generation since admixture event) (Geza et al., 2018) and a recently developed unified interface should help the user to select appropriate tools based on the data and application under consideration (Geza et al., 2020).

Introgression of genetic variation from a donor species into a recipient can provide adaptive advantages (i.e. adaptive introgression). The transfer of advantageous pre-adapted alleles from one species into another removes the reliance of new traits arising through de novo mutation in the recipient; the most famous example in invasive insect populations is the transfer of a large inversion conferring higher resistance to stress (desiccation, immunity genes) and insecticide resistance genes in *Anopheles* mosquitoes (Mendes et al., 2010; Fouet et al., 2012; Clarkson et al., 2014). Machine learning approaches to detect adaptive introgression from genome-scans are currently under development; such as the kNN technique implemented in the R package PopGenome (Pfeifer et al., 2014; 2020).

**5. Landscape genetics**

Landscape genetics approaches allow to determine the local factors shaping the spatial distribution of genetic variability (Manel et al., 2003; Storfer et al., 2010) and should provide a better understanding of the role of dispersal in the expansion process of invasive populations. The relative role of active versus passive dispersal must be analyzed at the landscape scale in expanding edges that are part of the same introduction event (colonization routes). Because spatial patterns of genetic variation can confound with population demographic history (Epps & Keyghobadi, 2015), it is important to have a good knowledge of the demographic changes during introduction before attempting to describe post-introduction expansion processes (demographic inferences).

Based on the causal relationship between dispersal and gene flow, the genetic structure of populations has been used to quantify the effective dispersal across populations. The isolation by distance hypothesis predicts a straight-line relationship between genetic and geographical distances that corresponds to the balance between processes promoting local population differentiation (mutation, drift) and homogenization (migration). However, landscape features can induce strong barriers to gene flow (i.e. isolation by barriers). The genetic variation among populations separated by a geographical barrier is expected to present a shift near a barrier (higher dissimilarities than expected under pure isolation by distance). Several methods have been developed to test for the presence of barriers, such as Geneland (Guillot et al., 2005), EEMS (Petkova et al., 2016), LocalDiff (Duforet-Frebourg & Blum, 2014), and SPLATCHE (Currat et al., 2004, 2019), which provided useful information concerning the spatial patterns of genetic variation at various spatial scales in *A. albopictus* European invasion (Sherpa et al., 2019a; Sherpa et al., 2020). However, these methods do not take explicitly into account the landscape features that separate gene pools, and barriers are deduced only from spatial patterns in genetic dissimilarities.

Recent approaches aim at quantifying the distance between populations according to the landscape features (see main text Table 2). These effective distances are calculated from resistance surfaces ​​that represent landscape impacts on the movement of organisms through space (Spear et al., 2010). Least-cost path models identify the shortest and single suitable path between two localities whereas multiple shortest paths models allow multiple dispersal routes (Pinto & Keitt, 2009). The distance along these paths can be calculated using commuteDistance in gdistance in R (van Etten, 2012) or CostDistance in ArcGIS (ESRI). More recently, circuit models also integrate multiple pairwise connections (McRae & Beier, 2007), such as implemented in Circuitscape (Shah & McRae, 2008). Multiple shortest paths and circuit models are probably the most representative of individual dispersal behavior in a heterogeneous landscape. The calculated resistance distances will depend of the raster properties used to build resistance surfaces, based on landscape heterogeneity and biological knowledge on the studied species. The grain size should be equal or smaller than natural dispersal capabilities. The resistance cost of each land-cover type can be arbitrary defined by the user (e.g. Medley et al., 2015), or without any a priori by testing each land-cover type as a barrier or corridor for gene flow (e.g. Sherpa et al., 2020). These costs can be further adjusted to the neighborhood based on the assumption that aggregated patches of suitable habitat may facilitate higher rates of gene flow.

The relationship between genetic distances and geographic or resistance distances, can then be estimated by Mantel tests or multiple regression tests between distance matrices (see main Table 2). While Mantel tests are limited to one response and two predictor matrices, multiple regressions can evaluate the effects of multiple predictors and their relative importance. Furthermore, Mantel tests show inflated type I errors compared to multiple regression tests (Raufaste & Rousset, 2001; Balkenhol et al., 2009). More recent methods allow building composite resistance surfaces to calculate an overall resistance distance matrix rather than modelling separately the resistance of each surface (Peterman, 2014), also allowing for model selection using decision criterion (i.e. AIC; but see Franckowiak et al., 2017 for a discussion on biases in model selection).

**References**

1. Ahrens, C. W., Rymer, P. D., Stow, A., Bragg, J., Dillon, S., … Dudaniec, R. Y. (2018). The search for loci under selection: trends, biases and progress. *Molecular Ecology*, *27*, 1342–1356. https://doi.org/10.1111/mec.14549
2. Alexander, D. H., Novembre, J., & Lange, K. (2009). Fast model-based estimation of ancestry in unrelated individuals. *Genome Research*, *19*, 1655–1664. https://doi.org/10.1101/gr.094052.109
3. Balkenhol, N., Gugerli, F., Cushman, S. A., Waits, L. P., Coulon, A., ... Wagner, H. H. (2009). Identifying future research needs in landscape genetics: where to from here? *Landscape Ecology*, *24*, 455. https://doi.org/10.1007/s10980-009-9334-z
4. Beichman, A. C., Huerta-Sanchez, E., & Lohmueller, K. E. (2018). Using genomic data to infer historic population dynamics of nonmodel organisms. *Annual Review of Ecology, Evolution, and Systematics*. *49*, 433–456. https://doi.org/10.1146/annurev-ecolsys-110617-062431
5. Bittner, T. D., Hajek, A. E., Haavik, L., Allison, J., & Nahrung, H. (2017). Multiple introductions of *Sirex noctilio* (Hymenoptera: Siricidae) in northeastern North America based on microsatellite genotypes, and implications for biological control. *Biological Invasions*, *19*, 1431–1447. https://doi.org/10.1007/s10530-016-1365-1
6. Boitard, S., Rodriguez, W., Jay, F., Mona, S., & Austerlitz, F. (2016). Inferring population size history from large samples of genome-wide molecular data-an approximate Bayesian computation approach. *PLoS Genetics*, *12*, e1005877. https://doi.org/10.1371/journal.pgen.1005877
7. Brisbin, A., Bryc, K., Byrnes, J., Zakharia, F., Omberg, L., Degenhardt, J., ... & Bustamante, C. D. (2012). PCAdmix: principal components-based assignment of ancestry along each chromosome in individuals with admixed ancestry from two or more populations. *Human Biology*, *84*, 343–364. https://doi.org/10.3378/027.084.0401
8. Capblancq, T., Luu, K., Blum, M. G., & Bazin, E. (2018). Evaluation of redundancy analysis to identify signatures of local adaptation. *Molecular Ecology Resources*, *18*, 1223–1233. https://doi.org/10.1111/1755-0998.12906
9. Chikhi, L., Sousa, V. C., Luisi, P., Goossens, B., & Beaumont, M. A. (2010). The confounding effects of population structure, genetic diversity and the sampling scheme on the detection and quantification of population size changes. *Genetics*, *186*, 983–995. https://doi.org/10.1534/genetics.110.118661
10. Ciosi, M., Miller, N. J., Kim, K. S., Giordano, R., Estoup, A., & Guillemaud, T. (2008). Invasion of Europe by the western corn rootworm, *Diabrotica virgifera virgifera*: multiple transatlantic introductions with various reductions of genetic diversity. *Molecular Ecology*, *17*, 3614–3627. https://doi.org/10.1111/j.1365-294X.2008.03866.x
11. Clarkson, C. S., Weetman, D., Essandoh, J., Yawson, A. E., Maslen, G., Manske, M., ... & Donnelly, M. J. (2014). Adaptive introgression between *Anopheles* sibling species eliminates a major genomic island but not reproductive isolation. *Nature Communications*, *5*, 1–10. https://doi.org/10.1038/ncomms5248
12. Cornuet, J. M., Piry, S., Luikart, G., Estoup, A., & Solignac, M. (1999). New methods employing multilocus genotypes to select or exclude populations as origins of individuals. *Genetics*, *153*, 1989–2000.
13. Cornuet, J. M., Ravigné, V., & Estoup, A. (2010). Inference on population history and model checking using DNA sequence and microsatellite data with the software DIYABC (v1.0). *BMC Bioinformatics*, *11*, 401. https://doi.org/10.1186/1471-2105-11-401
14. Cornuet, J. M., Santos, F., Beaumont, M. A., Robert, C. P., Marin, J. M., ... Estoup, A. (2008). Inferring population history with DIYABC: a user-friendly approach to approximate Bayesian computation. *Bioinformatics*, *24*, 2713–2719. https://doi.org/10.1093/bioinformatics
15. Currat, M., Arenas, M., Quilodran, C.S., Excoffier, L., & Ray, N. (2019). SPLATCHE3: simulation of serial genetic data under spatially explicit evolutionary scenarios including long-distance dispersal. *Bioinformatics, 35*, 4480–4483.
16. Currat, M., Ray, N., & Excoffier, L. (2004). SPLATCHE: a program to simulate genetic diversity taking into account environmental heterogeneity. *Molecular Ecology Notes*, *4*, 139–142. https://doi.org/10.1046/j.1471-8286.2003.00582.x
17. De Villemereuil, P., & Gaggiotti, O. E. (2015). A new FST‐based method to uncover local adaptation using environmental variables. *Methods in Ecology and Evolution*, *6*, 1248–1258. https://doi.org/10.1111/2041-210X.12418
18. De Villemereuil, P., Frichot, É., Bazin, É., François, O., & Gaggiotti, O. E. (2014). Genome scan methods against more complex models: when and how much should we trust them? *Molecular Ecology*, *23*, 2006–2019. https://doi.org/10.1111/mec.12705
19. Dias-Alves, T., Mairal, J., & Blum, M. G. (2018). Loter: a software package to infer local ancestry for a wide range of species. *Molecular Biology and Evolution*, *35*, 2318–2326. https://doi.org/10.1093/molbev/msy126
20. Duforet-Frebourg, N., & Blum, M.G.B. (2014). Non-stationary patterns of isolation-by-distance: inferring measures of local genetic differentiation with Bayesian kriging. *Evolution, 68*, 1110–1123. https://doi.org/10.1111/evo.12342
21. Epps, C. W., & Keyghobadi, N. (2015). Landscape genetics in a changing world: disentangling historical and contemporary influences and inferring change. *Molecular Ecology*, *24*, 6021–6040. https://doi.org/10.1111/mec.13454
22. Estoup, A., & Guillemaud, T. (2010). Reconstructing routes of invasion using genetic data: why, how and so what? *Molecular Ecology*, *19*, 4113–4130. https://doi.org/10.1111/j.1365-294X.2010.04773.x
23. Excoffier, L., & Heckel, G. (2006). Computer programs for population genetics data analysis: a survival guide. *Nature Reviews Genetics*, *7*, 745–758. https://doi.org/10.1038/nrg1904
24. Facon, B., Crespin, L., Loiseau, A., Lombaert, E., Magro, A., & Estoup, A. (2011). Can things get worse when an invasive species hybridizes? The harlequin ladybird *Harmonia axyridis* in France as a case study. *Evolutionary Applications*, *4*, 71–88. https://doi.org/10.1111/j.1752-4571.2010.00134.x
25. Fitzpatrick, B. M., Fordyce, J. A., Niemiller, M. L., & Reynolds, R. G. (2012). What can DNA tell us about biological invasions? *Biological Invasions*, *14*, 245–253. https://doi.org/10.1007/s10530-011-0064-1
26. Fitzpatrick, M. C., & Keller, S. R. (2015). Ecological genomics meets community‐level modelling of biodiversity: Mapping the genomic landscape of current and future environmental adaptation. *Ecology Letters*, *18*, 1–16. https://doi.org/10.1111/ele.12376
27. Foll, M., & Gaggiotti, O. (2008). A genome-scan method to identify selected loci appropriate for both dominant and codominant markers: a Bayesian perspective. *Genetics*, *180*, 977–993. https://doi.org/10.1534/genetics.108.092221
28. Fouet, C., Gray, E., Besansky, N. J., & Costantini, C. (2012). Adaptation to aridity in the malaria mosquito *Anopheles gambiae*: chromosomal inversion polymorphism and body size influence resistance to desiccation. *PloS one*, *7*, e34841. https://doi.org/10.1371/journal.pone.0034841
29. Franckowiak, R. P., Panasci, M., Jarvis, K. J., Acuna-Rodriguez, I. S., Landguth, E. L., … Wagner, H. H. (2017). Model selection with multiple regression on distance matrices leads to incorrect inferences. *PloS One*, *12*, e0175194. https://doi.org/10.1371/journal.pone.0175194
30. Frichot, E., Schoville, S. D., Bouchard, G., & François, O. (2013). Testing for associations between loci and environmental gradients using latent factor mixed models. *Molecular Biology and Evolution*, *30*, 1687–1699. https://doi.org/10.1093/molbev/mst063
31. Gautier, M. (2015). Genome-wide scan for adaptive divergence and association with population-specific covariates. *Genetics*, *201*, 1555–1579. https://doi.org/10.1534/genetics.115.181453
32. Geneva, A. J., Muirhead, C. A., Kingan, S. B., & Garrigan, D. (2015). A new method to scan genomes for introgression in a secondary contact model. *PloS One*, *10*, e0118621. https://doi.org/10.1371/journal.pone.0118621
33. Geza, E., Mugo, J., Mulder, N. J., Wonkam, A., Chimusa, E. R., & Mazandu, G. K. (2019). A comprehensive survey of models for dissecting local ancestry deconvolution in human genome. *Briefings in Bioinformatics*, *20*, 1709–1724. https://doi.org/10.1093/bib/bby044
34. Geza, E., Mulder, N. J., Chimusa, E. R., & Mazandu, G. K. (2020). FRANC: a unified framework for multi-way local ancestry deconvolution with high density SNP data. *Briefings in Bioinformatics*, *21*, 1837–1845. https://doi.org/10.1093/bib/bbz117
35. Guillemaud, T., Blin, A., Le Goff, I., Desneux, N., Reyes, M., … Lombaert, E. (2015). The tomato borer, *Tuta absoluta*, invading the Mediterranean Basin, originates from a single introduction from Central Chile. *Scientific Reports*, *5*, 8371. https://doi.org/10.1038/srep08371
36. Guillot, G., Mortier, F., & Estoup, A. (2005). GENELAND: a computer package for landscape genetics. *Molecular Ecology Notes*, *5*, 712–715. https://doi.org/10.1111/j.1471-8286.2005.01031.x
37. Hohenlohe, P. A., Phillips, P. C., & Cresko, W. A. (2010). Using population genomics to detect selection in natural populations: key concepts and methodological considerations. *International Journal of Plant Sciences*, *171*, 1059–1071. https://doi.org/10.1086/656306
38. Kerdelhué, C., Boivin, T., & Burban, C. (2014). Contrasted invasion processes imprint the genetic structure of an invasive scale insect across southern Europe. *Heredity*, *113*, 390–400. https://doi.org/10.1038/hdy.2014.39
39. Li, H., & Durbin, R. (2011). Inference of human population history from individual whole-genome sequences. *Nature*, *475*, 493–496. https://doi.org/10.1038/nature10231
40. Liu, X., & Fu, Y. X. (2015). Exploring population size changes using SNP frequency spectra. *Nature Genetics*, *47*, 555–559. https://doi.org/10.1038/ng.3254
41. Lombaert, E., Guillemaud, T., Lundgren, J., Koch, R., Facon, B., ... Staverlokk, A. (2014). Complementarity of statistical treatments to reconstruct worldwide routes of invasion: the case of the Asian ladybird *Harmonia axyridis*. *Molecular Ecology*, *23*, 5979–5997. https://doi.org/10.1111/mec.12989
42. Luu, K., Bazin, E., & Blum, M.G.B., (2017). pcadapt: an R package to perform genome scans for selection based on principal component analysis. *Molecular Ecology Resources,* *1,* 67–77. https://doi.org/10.1111/1755-0998.12592
43. Manel, S., Schwartz, M. K., Luikart, G., & Taberlet, P. (2003). Landscape genetics: combining landscape ecology and population genetics. *Trends in ecology & evolution*, *18*, 189–197. https://doi.org/10.1016/S0169-5347(03)00008-9
44. Manion, G., Lisk, M., Ferrier, S., Nieto‐Lugilde, D., Mokany, K., & Fitzpatrick, M. C. (2017). gdm: Generalized Dissimilarity Modeling. R package version 1.3.11. https://CRAN.R-project.org/ package=gdm
45. Maples, B. K., Gravel, S., Kenny, E. E., & Bustamante, C. D. (2013). RFMix: a discriminative modeling approach for rapid and robust local-ancestry inference. *The American Journal of Human Genetics*, *93*, 278–288. https://doi.org/10.1016/j.ajhg.2013.06.020
46. Martin, S. H., Davey, J. W., & Jiggins, C. D. (2015). Evaluating the use of ABBA–BABA statistics to locate introgressed loci. *Molecular Biology and Evolution*, *32*, 244–257. https://doi.org/10.1093/molbev/msu269
47. McRae, B. H., & Beier, P. (2007). Circuit theory predicts gene flow in plant and animal populations. *Proceedings of the National Academy of Sciences*, *104*, 19885–19890. https://doi.org/10.1073/pnas.0706568104
48. Medley, K. A., Jenkins, D. G., & Hoffman, E. A. (2015). Human‐aided and natural dispersal drive gene flow across the range of an invasive mosquito. *Molecular Ecology*, *24*, 284–295. https://doi.org/10.1111/mec.12925
49. Mendes, C., Felix, R., Sousa, A. M., Lamego, J., Charlwood, D., Do Rosário, V. E., ... & Silveira, H. (2010). Molecular evolution of the three short PGRPs of the malaria vectors *Anopheles gambiae* and *Anopheles arabiensis* in East Africa. *BMC Evolutionary Biology*, *10*, 1–12. https://doi.org/10.1186/1471-2148-10-9
50. Mussmann, S. M., Douglas, M. R., Bangs, M. R., & Douglas, M. E. (2019). Comp-D: a program for comprehensive computation of D-statistics and population summaries of reticulated evolution. *Conservation Genetics Resources*, *12*, 263–267. https://doi.org/10.1007/s12686-019-01087-x
51. Paşaniuc, B., Sankararaman, S., Kimmel, G., & Halperin, E. (2009). Inference of locus-specific ancestry in closely related populations. *Bioinformatics*, *25*, 213–221. https://doi.org/10.1093/bioinformatics/btp197
52. Patterson, N., Moorjani, P., Luo, Y., Mallick, S., Rohland, N., Zhan, Y., ... & Reich, D. (2012). Ancient admixture in human history. *Genetics*, *192*, 1065–1093. https://doi.org/10.1534/genetics.112.145037
53. Peery, M. Z., Kirby, R., Reid, B. N., Stoelting, R., Doucet-Bëer, E. L. E. N. A., ... Palsbøll, P. J. (2012). Reliability of genetic bottleneck tests for detecting recent population declines. *Molecular Ecology*, *21*, 3403–3418. https://doi.org/10.1111/j.1365-294X.2012.05635.x
54. Peterman, W. E. (2014). ResistanceGA: An R package for the optimization of resistance surfaces using genetic algorithms.
55. Petkova, D., Novembre, J., & Stephens, M. (2016). Visualizing spatial population structure with estimated effective migration surfaces. *Nature Genetics*, *48*, 94. https://doi.org/10.1038/ng.3464
56. Pfeifer, B., & Kapan, D. D. (2019). Estimates of introgression as a function of pairwise distances. *BMC Bioinformatics*, *20*, 1–11. https://doi.org/10.1186/s12859-019-2747-z
57. Pfeifer, B., Alachiotis, N., Pavlidis, P., & Schimek, M. G. (2020). Genome scans for selection and introgression based on k‐nearest neighbour techniques. *Molecular Ecology Resources*, *20*, 1597–1609. https://doi.org/10.1111/1755-0998.13221
58. Pfeifer, B., Wittelsbürger, U., Ramos-Onsins, S. E., & Lercher, M. J. (2014). PopGenome: an efficient Swiss army knife for population genomic analyses in R. *Molecular Biology and Evolution*, *31*, 1929–1936. https://doi.org/10.1093/molbev/msu136
59. Pickrell, J., & Pritchard, J. (2012). Inference of population splits and mixtures from genome-wide allele frequency data. *Nature Precedings*, 1–1. https://doi.org/10.1038/npre.2012.6956.1
60. Pinto, N., & Keitt, T. H. (2009). Beyond the least-cost path: evaluating corridor redundancy using a graph-theoretic approach. *Landscape Ecology*, *24*, 253–266. https://doi.org/10.1007/s10980-008-9303-y
61. Piry, S., Alapetite, A., Cornuet, J. M., Paetkau, D., Baudouin, L., & Estoup, A. (2004). GENECLASS2: a software for genetic assignment and first-generation migrant detection. *Journal of Heredity*, *95*, 536–539. https://doi.org/10.1093/jhered
62. Piry, S., Luikart, G., & Cornuet, J. M. (1999). BOTTLENECK: a program for detecting recent effective population size reductions from allele data frequencies. *Journal of Heredity*, *90*, 502–503. https://doi.org/10.1093/jhered/90.4.502
63. Price, A. L., Tandon, A., Patterson, N., Barnes, K. C., Rafaels, N., Ruczinski, I., ... & Myers, S. (2009). Sensitive detection of chromosomal segments of distinct ancestry in admixed populations. *PLoS Genetics*, *5*, e1000519. https://doi.org/10.1371/journal.pgen.1000519
64. Pritchard, J. K., Stephens, M., & Donnelly, P. (2000). Inference of population structure using multilocus genotype data. *Genetics*, *155*, 945–959.
65. Racimo, F., Sankararaman, S., Nielsen, R., & Huerta-Sánchez, E. (2015). Evidence for archaic adaptive introgression in humans. *Nature Reviews Genetics*, *16*, 359–371. https://doi.org/10.1038/nrg3936
66. Rannala, B., & Mountain, J. L. (1997). Detecting immigration by using multilocus genotypes. *Proceedings of the National Academy of Sciences*, *94*, 9197–9201. https://doi.org/10.1073/pnas.94.17.9197
67. Raufaste, N., & Rousset, F. (2001). Are partial Mantel tests adequate? *Evolution*, *55*, 1703–1705. https://doi.org/10.1111/j.0014-3820.2001.tb00689.x
68. Rellstab, C., Gugerli, F., Eckert, A. J., Hancock, A. M., & Holderegger, R. (2015). A practical guide to environmental association analysis in landscape genomics. *Molecular Ecology*, *24*, 4348–4370. https://doi.org/10.1111/mec.13322
69. Robert, C. P., Cornuet, J. M., Marin, J. M., & Pillai, N. S. (2011). Lack of confidence in approximate Bayesian computation model choice. *Proceedings of the National Academy of Sciences*, *108*, 15112–15117. https://doi.org/10.1073/pnas.1102900108
70. Rosenzweig, B. K., Pease, J. B., Besansky, N. J., & Hahn, M. W. (2016). Powerful methods for detecting introgressed regions from population genomic data. *Molecular Ecology*, *25*, 2387–2397. <https://doi.org/10.1111/mec.13610>
71. Rozas, J., Sánchez-DelBarrio, J. C., Messeguer, X., & Rozas, R. (2003). DnaSP, DNA polymorphism analyses by the coalescent and other methods. *Bioinformatics*, *19*, 2496–2497. https://doi.org/10.1093/bioinformatics/btg359
72. Salmona, J., Heller, R., Lascoux, M., & Shafer, A. (2017). Inferring demographic history using genomic data. In *Population Genomics: Concepts, Approaches and Applications.* Ed Rajora, O. P.*,* pp. 511–537. Springer.
73. Schiffels, S., & Durbin, R. (2014). Inferring human population size and separation history from multiple genome sequences. *Nature Genetics*, *46*, 919–925. https://doi.org/10.1038/ng.3015
74. Shah, V. B., & McRae, B. H. (2008). CIRCUITSCAPE: a tool for landscape ecology. In *Proceedings of the 7th Python in Science Conference* pp. 62–66. Pasadena, California: SciPy. https://doi.org/10.1890/07-1861.1
75. Sherpa, S., Blum, M. G., & Després, L. (2019b). Cold adaptation in the Asian tiger mosquito's native range precedes its invasion success in temperate regions. *Evolution*, *73*, 1793–1808. https://doi.org/10.1111/evo.13801
76. Sherpa, S., Blum, M. G., Capblancq, T., Cumer, T., Rioux, D., & Després, L. (2019a). Unravelling the invasion history of the Asian tiger mosquito in Europe. *Molecular Ecology*, *28*, 2360–2377. https://doi.org/10.1111/mec.15071
77. Sherpa, S., Guéguen, M., Renaud, J., Blum, M. G., Gaude, T., ... & Després, L. (2019c). Predicting the success of an invader: Niche shift versus niche conservatism. *Ecology and Evolution*, *9*, 12658–12675. https://doi.org/10.1002/ece3.5734
78. Sherpa, S., Renaud, J., Guéguen, M., Besnard, G., Mouyon, L., … Després, L. (2020). Landscape does matter: disentangling founder effects from natural and human-aided post-introduction dispersal during an ongoing biological invasion. *Evolutionary Applications*, In press.
79. Sherpa, S., Rioux, D., Goindin, D., Fouque, F., François, O., & Despres, L. (2018). At the origin of a worldwide invasion: unraveling the genetic makeup of the Caribbean bridgehead populations of the dengue vector *Aedes aegypti.* *Genome Biology and Evolution*, *10*, 56–71. https://doi.org/10.1093/gbe/evx267
80. Spear, S. F., Balkenhol, N., Fortin, M. J., McRae, B. H., & Scribner, K. I. M. (2010). Use of resistance surfaces for landscape genetic studies: considerations for parameterization and analysis. *Molecular Ecology*, *19*, 3576–3591. https://doi.org/10.1111/j.1365-294X.2010.04657.x
81. Städler, T., Haubold, B., Merino, C., Stephan, W., & Pfaffelhuber, P. (2009). The impact of sampling schemes on the site frequency spectrum in nonequilibrium subdivided populations. *Genetics*, *182*, 205–216. https://doi.org/10.1534/genetics.108.094904
82. Storfer, A., Murphy, M. A., Spear, S. F., Holderegger, R., & Waits, L. P. (2010). Landscape genetics: where are we now? *Molecular Ecology*, *19*, 3496–3514. https://doi.org/10.1111/j.1365-294X.2010.04691.x
83. Terhorst, J., Kamm, J. A., & Song, Y. S. (2017). Robust and scalable inference of population history from hundreds of unphased whole genomes. *Nature Genetics*, *49*, 303. https://doi.org/10.1038/ng.3748
84. van Etten, J. (2012). gdistance: Distances and routes on geographical grids. R package.
85. Whitlock, M. C., & Lotterhos, K. E. (2015). Reliable detection of loci responsible for local adaptation: inference of a null model through trimming the distribution of FST. *The American Naturalist*, *186*, S24–S36. https://doi.org/10.1111/mec.13100
86. Yang, J. J., Li, J., Buu, A., & Williams, L. K. (2013). Efficient inference of local ancestry. *Bioinformatics*, *29*, 2750–2756. https://doi.org/10.1093/bioinformatics/btt488
